# Prenatal Detection of *HSPG2*‐Related Dyssegmental Dysplasia, Silverman–Handmaker Type: A Rare Case Report

**DOI:** 10.1155/crog/5094018

**Published:** 2026-10-07

**Authors:** Omayyah Dar-Odeh, Raghad Abdeljawad, Rahaf Abdeljawad, Najla Dar-Odeh

**Affiliations:** ^1^ Obstetrics and Gynecology Department Arab Medical Center Amman Jordan; ^2^ School of Medicine Hashemite University Zarqa Jordan hu.edu.jo; ^3^ School of Dentistry The University of Jordan Amman Jordan ju.edu.jo

**Keywords:** case report, dyssegmental dysplasia, heparan sulfate proteoglycan of basement membrane, *HSPG2*, skeletal dysplasia

## Abstract

**Introduction:**

*HSPG2*‐related dyssegmental dysplasia (heparan sulfate proteoglycan 2) is an extremely rare and severe autosomal recessive skeletal dysplasia. The low incidence and the overlapping features with other types of lethal skeletal dysplasia represent the main challenges in diagnosis. Early diagnosis is essential and can be achieved by ultrasound examination and genetic analysis. This report describes a case of *HSPG2*‐related dyssegmental dysplasia, Silverman–Handmaker type (DDSH) diagnosed prenatally through a combination of detailed ultrasound imaging and subsequent genetic analysis.

**Case Presentation:**

A 21‐year‐old pregnant lady (G2P1) was referred to our center at 17‐week gestation upon the detection of severe shortening of fetal limbs. Detailed ultrasound examination revealed small for gestational age, and specific craniofacial features such as severe micrognathia, short neck, microcephaly, prominent eyes, and occipital encephalocele. Other abnormalities were detected in extremities in the form of bowing of lower limbs, severe tetramicromelia, and upper limbs in a fixed flexion. Amniocentesis for whole exome sequencing was performed. It showed a homozygous pathogenic variant in the *HSPG2* gene (c.11565+1G>A), a result consistent with the genetic diagnosis of autosomal recessive *HSPG2*‐related DDSH. Medical termination was performed based on the decision of parents. Parents signed a written consent for the publication of this case report.

**Discussion:**

Findings of this report support the growing evidence of the phenotypic spectrum and genetic underpinnings of the lethal disorder of *HSPG2*‐related DDSH, emphasizing the importance of early and accurate diagnosis for optimal patient care. DDSH can be diagnosed prenatally using fetal ultrasound as early as 13 weeks. Radiographic assessment and molecular analysis of the *HSPG2* gene are essential for confirming the prenatal diagnosis. Accurate diagnosis enables appropriate healthcare delivery and facilitates informed reproductive decision‐making, including prenatal diagnosis and preimplantation genetic testing in future pregnancies.

## 1. Introduction

*HSPG2*‐related dyssegmental dysplasia is an exceptionally rare and severe autosomal recessive skeletal dysplasia. On the other hand, the spectrum of *HSPG2*‐related skeletal dysplasias includes dyssegmental dysplasia (Silverman–Handmaker and Rolland–Desbuquois types) and Schwartz–Jampel syndrome (SJS); each characterized by distinct clinical features and prognostic outcomes. The mildest form of these is represented by SJS, which is characterized by myotonia, chondrodysplasia, and a characteristic mask‐like facial appearance. Owing to its relatively mild manifestations, affected individuals generally have a normal life expectancy [1].

*HSPG2* itself is an essential, highly conserved gene whose expression influences many developmental processes including the formation of the heart and brain by encoding perlecan. The gene is widely expressed throughout the musculoskeletal system including cartilage, bone marrow and skeletal muscle, and is involved in angiogenesis and cellular signaling [2].

*HSPG2*‐related dyssegmental dysplasia, Silverman–Handmaker type (DDSH) is a rare syndrome with a reported incidence of less than 1 in 1,000,000 [3]. Less than 40 cases and 10 different pathogenic *HSPG2* variants have been reported so far in literature [4].

Features of DDSH are caused by functional null variants in *HSPG2*. Severe generalized anisospondyly with defective vertebral ossification and micromelia are important characteristics. Literature reports distinctive radiographic findings such as generalized anisospondyly (vertebral bodies of irregular size, shape, and ossification), marked platyspondyly (defective vertebral segmentation), and kyphoscoliotic deformities [5]. Other radiographic features are detected in the long bones which are severely shortened and bowed, with metaphyseal flaring and irregular ossification. Narrow thorax, short ribs, pelvic hypoplasia, highly reduced joint mobility, and shortened tubular bones of the hands and feet may also be present [6, 7]. Further, characteristic craniofacial features are reported such as flat face, micrognathia, cleft palate, and encephalocele. On the other hand, extraskeletal features include genitourinary, cardiovascular [8], and pulmonary abnormalities. Pulmonary hypoplasia is associated with short, broad, and anteriorly cupped ribs, whereas the scapulae appear short with hypoplastic glenoids [3]. Joint contractures, metatarsus varus, and pes equinovarus may also be present [9]. The disorder outcome is usually lethal leading to intrauterine death or short‐term postnatal survival [6], that may last up to 8 months [10].

Prenatal diagnosis of DDSH is challenging due to its low incidence and the overlapping features with other lethal types of skeletal dysplasia. So far, only eight cases were diagnosed prenatally We present a case of DDSH diagnosed prenatally using detailed fetal ultrasound and confirmatory genetic analysis, which identified an *HSPG2* variant that, to the best of our knowledge, has not previously been reported in association with DDSH.

## 2. Case Report

A 21‐year‐old pregnant lady (G2P1) was referred to our center at 17‐week gestation due to the detection of severe shortening of fetal limbs. She was in a consanguineous marriage, and her medical history showed that she previously had a healthy child. She was a nonsmoker and reported no chronic diseases and no medication intake. There was no family history of similar abnormalities.

### 2.1. Ultrasound Examination

Detailed ultrasound examination (Samsung HS70, South Korea) was conducted. It revealed small for gestational age, severe micrognathia, short neck, microcephaly and occipital encephalocele (Figure 1), upper limbs in a fixed flexion, bowing of lower limbs (camptomelia), severe tetramicromelia (bilateral severe rizomeso‐acromelic shortening of upper and lower limbs), disorganized vertebral bodies (Figure 2), prominent eyes (Figure 3), talipes equinovarus and skin edema (Figure 4). Fetal biometric measurements were taken as described in Table 1.

**Figure 1:**
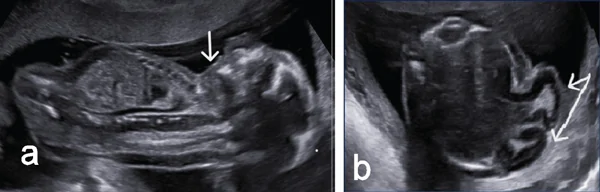
Craniofacial features as presented by ultrasound examination. Arrows point to (a) severe micrognathia and (b) occipital encephalocele.

**Figure 2:**
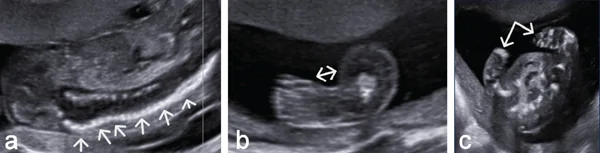
Ultrasonographic features of extremities. Arrows point to (a) disorganized vertebral bodies, (b) bowing of lower limbs, and (c) upper arms in fixed flexion.

**Figure 3:**
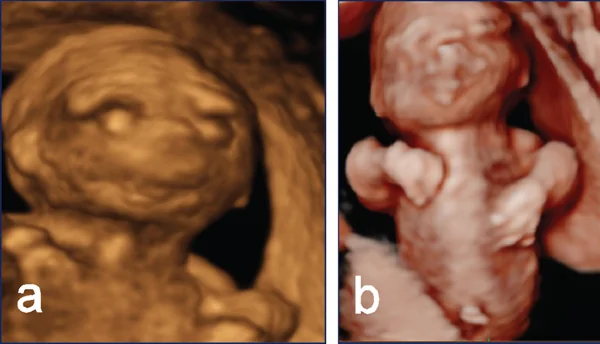
3D image of the fetus showing (a) prominent eyes and (b) fixed flexion of the upper limbs.

**Figure 4:**
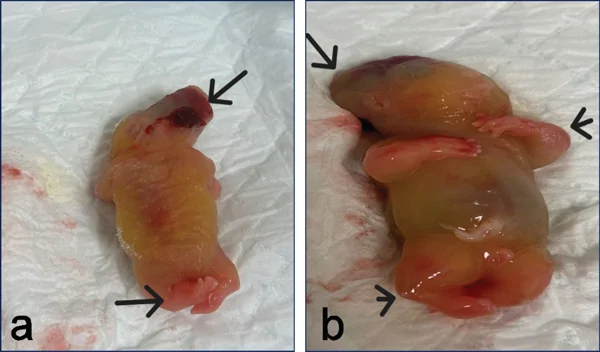
Postnatal pictures of the abortus. Arrows point to (a) micrognathia, facial dysmorphism very short neck equinovarus deformity and (b) flexed short upper and lower limbs, and occipital encephalocele.

**Table 1:** Fetal biometric measurements at 17‐week gestational age.

Fetal parameters	Measurement	Percentile
Femur length	0.91 cm	1st
Head circumference	10.12 cm	1st
Abdominal circumference	11.67 cm	64th
Humerus length	0.95 cm	1st
Tibia length	0.55 cm	1st
Fibula length	0.6 cm	4th
Estimated fetal weight	117 g	< 1st

A preliminary diagnosis of skeletal dysplasia was made. The parents were counseled regarding severity of the abnormalities and the need for further genetic studies. Therefore, amniocentesis for whole exome sequencing (WES) was performed.

### 2.2. Genetic Analysis

Genomic DNA was enzymatically fragmented, and target regions were enriched using DNA capture probes. The generated library was sequenced on an Illumina platform to achieve a minimum coverage depth of 20x for > 98% of the targeted bases. An in‐house bioinformatics pipeline was employed, including read alignment to GRCh37/hg19 genome assembly, variant calling, annotation, and comprehensive variant filtering. Variants with minor allele frequency (MAF) < 1*%* in gnomAD database, as well as the previously reported disease‐causing variants listed in HGMD, ClinVar or CentoMD, were evaluated. Variant analysis focused on coding exons and flanking ± 10 intronic nucleotides of genes with a clear gene‐phenotype associations (based on OMIM information). All potential inheritance patterns were considered. Family history and clinical information were incorporated into the assessment of variant pathogenicity and disease causality. Variants were classified into five categories (pathogenic, likely pathogenic, variant of uncertain significance [VUS], likely benign, and benign) according to ACMG/AMP guidelines for sequence variant interpretation [11]. All clinically relevant variants associated with the patient′s phenotype were reported.

CENTOGENE has established stringent quality control and validation procedures for variants detected by next‐generation sequencing (NGS). Variants with low sequencing quality and/or uncertain zygosity were confirmed by orthogonal methods, resulting in a reported specificity exceeding 99.9% for all reported variants. To exclude maternal cell contamination (MCC), 15 autosomal short tandem repeat (STR) markers and amelogenin were analyzed using the PowerPlex 16HS multiplex kit (Promega Corporation, Madison, Wisconsin, United States). Given the prenatal presentation, disease severity, and skeletal phenotype observed in this case, the clinical findings were considered most consistent with DDSH. Genetic analysis identified a homozygous variant in *HSPG2*, NM_001291860.1:c.11565+1G>A (dbSNP: rs2550621846); classified as pathogenic according to ACMG criteria.

The couple opted for termination of pregnancy; however, they declined fetal autopsy. Medical termination was performed at 19 weeks and 3 days gestation. External examination of abortus confirmed ultrasonographic features. The abortus showed facial dysmorphism, micrognathia, very short neck, flexed short extremities, equinovarus deformity and occipital encephalocele (Figure 4). A babygram was not performed, as the termination was carried out at another hospital. Parents were provided with comprehensive genetic counseling and advised to consider preimplantation genetic testing (PGT) to mitigate the associated risk. However, the couple declined this intervention and subsequently achieved a spontaneous conception. One year after delivery of this case, the mother gave birth to a completely healthy baby boy. Parents signed a written consent for the publication of this case report.

## 3. Discussion

This report is aimed at contributing to the limited literature on prenatal diagnosis of DDSH, highlighting the importance of meticulous ultrasonographic evaluation and the role of molecular testing in confirming diagnosis and guiding appropriate management. This is crucial for accurate counseling and informed decision‐making for affected families. Less than 40 cases of DD are reported so far in the literature. Approximately 10 cases were prenatally detected, among which eight were confirmed by molecular genetic analysis [7, 12, 6]. Studies reporting these cases and the associated *HSPG2* variants are presented in (Table 2).

**Table 2:** *HSPG2* variants that are reported so far in cases of *HSPG2*‐related dyssegmental dysplasia, Silverman–Handmaker type.

Author (year)	*HSPG2* variant
Arikawa‐Hirasawa et al. (2001) [5]	G.7086 + 5A (I 52);
	C.10328T (E 73)
Rieubland et al. (2010) [12]	c.3876–3879delGTGC
Basalom et al. (2018) [6]	c.4029+1G>A
Wang and Wang (2022) [13]	c.6001dupC (p. R2001pfs∗19) and c.11207G>A (p. R373Q)
Akcan et al. (2025) [2]	c.1355 + 1 G>T
Ladhani et al. (2013) [7]	c.646G>T (E 7); c.5788C>T
Vadukkuut et al. (2021) [14]	c.9541_9542delGG; (Gly3181HisfsTer36)
Present case	c.11565+1G>A

Similar to previous studies, the antenatal features of the current case were detected in the second trimester [6]. However, previous reports have documented prenatal detection of DDSH as early as 13 weeks of gestation [7]. Moreover, severe and typically lethal features may occasionally be identified during the first trimester [6].

The fetus exhibited several characteristic features previously described in DDSH, including severe tetramicromelia, camptomelia, micrognathia, vertebral segmentation abnormalities, fixed limb contractures, and talipes equinovarus. In addition, occipital encephalocele was observed, a finding that was previously described by a few reports [5, 12].

The molecular test of choice is WES. Here, we report a variant not previously described in association with DDSH, *HSPG2* variant c.11565+1G>A. The variant was classified as pathogenic according to the ACMG/AMP guidelines, and as refined by the ClinGen Sequence Variant Interpretation (SVI) Working Group. The phenotype was interpreted as being most consistent with DDSH, given its severe prenatal onset and predominant skeletal manifestations, including markedly shortened long bones.

Radiographic examination and DNA analysis of the *HSPG2* gene are necessary for confirming the diagnosis and for preimplantation and prenatal diagnosis in pregnancies at risk [7]. Given that this skeletal dysplasia is typically lethal and diagnosable via prenatal imaging, the immediate impact of molecular diagnosis on the outcome of the affected pregnancy may seem to be limited. However, establishing the molecular diagnosis is crucial for confirming autosomal recessive inheritance, excluding sporadic cases, and accurately defining the recurrence risk estimated to be 25%. This, in turn, enables more informed reproductive planning, including prenatal and PGT in future pregnancies.

Parents of this case were a consanguineous couple. A previous report described two married cousins who had a fetus with DDSH [15]. This indicates the importance of prenatal examination particularly in geographic areas where consanguineous marriages are common, which increases the probability that both parents will be carriers of the same recessive variant. This case also highlights several health risk behaviors that are frequently seen in geographic areas of low healthcare resources [16]. Parents declined to do any genetic study because of financial reasons as these tests were not covered by their health insurance plan. Due to limited financial resources, no karyotyping or chromosome microarray was conducted. Further, parental segregation testing was not performed. Parents declined the genetic counseling that was offered, and opted for spontaneous pregnancy, albeit their knowledge of the potential associated risks. It is important to engage families in the diagnostic process and treatment planning. Beyond establishing a definitive diagnosis, families require clear communication, psychological support, and accurate genetic counseling to understand the nature of the disorder, its prognosis, and recurrence risks. Effective counseling helps parents make informed reproductive decisions and reduces anxiety associated with uncertainty. The healthcare system plays a central role in ensuring timely access to multidisciplinary services, including clinical genetics, fetal medicine, pediatrics, and psychological support. Management often requires long‐term follow‐up, repeated diagnostic evaluations, specialized consultations, and in some cases costly interventions, all of which can impose a significant financial burden on families.

This report has limitations. Ultrasound has a limited accuracy of 67.9% in diagnosing skeletal dysplasias [17]. A babygram was not performed, and no measurements of the abortus were obtained, as the termination was carried out at another hospital. Furthermore, required genetic analysis including parental segregation testing, karyotyping or chromosome microarray was not performed. This could have compromised the estimation of recurrence risk and the detection of microdeletions or microduplications, with a subsequent overall diagnostic uncertainty.

## 4. Conclusions

Prenatal diagnosis of DDSH can be achieved using ultrasonographic examination and molecular genetic analysis. Utilization of these diagnostic methods is recommended particularly in some geographic areas with characteristic cultural backgrounds to allow for early diagnosis and timely counseling and planning.

## Author Contributions

Omayyah Dar‐Odeh: conceptualization, methods, writing, first draft; Raghad Abdeljawad: writing, first draft; Rahaf Abdeljawad and Najla Dar‐Odeh: writing, review and editing.

## Funding

No funding was received for this manuscript.

## Disclosure

All authors have read and approved the final version of the manuscript. The corresponding author had full access to all of the data in this study and takes complete responsibility for the integrity of the data and the accuracy of the data analysis. Najla Dar‐Odeh affirms that this manuscript is an honest, accurate, and transparent account of the study being reported; that no important aspects of the study have been omitted; and that any discrepancies from the study as planned (and, if relevant, registered) have been explained.

## Consent

A signed written consent was obtained from parents for the publication of this case report.

## Conflicts of Interest

The authors declare no conflicts of interest.

## Data Availability

The data that support the findings of this study are available on request from the corresponding author. The data are not publicly available due to privacy or ethical restrictions.
